# Failed treatment of long bone nonunions with low intensity pulsed ultrasound

**DOI:** 10.1007/s00402-016-2501-1

**Published:** 2016-07-06

**Authors:** Bahram Biglari, Timur Mert Yildirim, Tyler Swing, Thomas Bruckner, Wolfgang Danner, Arash Moghaddam

**Affiliations:** Department of Paraplegiology and Technical Orthopaedics, BG Trauma Centre Ludwigshafen, Ludwig-Guttmann-Str. 13, 67071 Ludwigshafen, Germany; HTRG-Heidelberg Trauma Research Group, Centre for Orthopaedics, Trauma Surgery and Spinal Cord Injury, Heidelberg University Hospital, Schlierbacher Landstraße 200a, 69118 Heidelberg, Baden-Württemberg Germany; Institute of Medical Biometry and Informatics, University of Heidelberg, Im Neuenheimer Feld 305, 69120 Heidelberg, Germany

**Keywords:** Nonunion, Delayed fracture healing, Low intensity pulsed ultrasound, LIPUS, EXOGEN^®^

## Abstract

**Introduction:**

The use of low intensity pulsed ultrasound (LIPUS) in the treatment of nonunions is still controversial. The present study is concerned with whether this procedure has a clinical use and which cofactors influence its therapeutic results.

**Methods:**

In this prospective, single institution, observational study, data from October 2010 to October 2013 from 61 nonunions in 60 patients treated with EXOGEN^®^ LIPUS therapy were analysed. The average age was 45.4 ± 9.81 (18–63) years. Treatment was primarily done on long bones of the lower extremity (75.4 %). All 61 nonunions were examined after treatment, and the rate of healing as well as functional and subjective results were evaluated. Based on clinical and radiological findings, patients were divided into two groups: G1—successful treatment; and G2—unsuccessful treatment. Groups were compared to one another to identify possible factors influencing treatment.

**Results:**

Twenty (32.8 %) patients showed bone consolidation with an average time of healing of 5.3 (2–7) months. In patients without successful treatment, who underwent revision surgery instead, full weight bearing took on average 3.7 months longer, and they were able to return to work 6.8 months later. Most of the treated patients (70.5 %) reported no improvement in pain. In G2, 12 (29.3 %) patients suffered in their previous history from osteitis; in G1 there were only two patients (10 %) (*p* = 0.012). There were further significant differences in the age of the fracture, the type of osteosynthesis, the gap size, as well as the NUSS score.

**Conclusion:**

Despite patients being chosen strictly according to EXOGEN^®^ indications, only a small number of patients with nonunions who underwent LIPUS therapy experienced successful treatment (32.8 %). Overall, its use resulted in a clear delay in the time of treatment, so that according to our results, the use of LIPUS should be seen critically in long bone nonunions and use should be made on a case-by-case basis.

## Introduction

Nonunion is often associated with pain and enormous reductions in the quality of life. Also, it is associated with longer disease duration, as well as social and economic consequences [1]. The point in time at which a fracture with delayed healing is considered a nonunion is controversial [2]. Increasing certainty exists over this definition of a nonunion: when bone consolidation is expected, but does not occur without intervention according to radiological and clinical results [3]. In order to treat nonunions there are invasive and non-invasive surgical therapies available. Non-invasive treatments, such as physiotherapy and mechanical weight bearing exercises [4, 5] are especially important in the early phase of nonunion treatment and require sufficient mechanical stability for possible osseous regeneration. If these methods fail, then surgical intervention gains importance [6–8]. In the realm of non-invasive therapy, there are further approaches with differing biophysical methods, such as high energy extracorporeal shock wave therapy [9, 10], pulsed electromagnetic field [11], or constant direct current [12], as well as therapy with low intensity pulsed ultrasound (LIPUS) [13–15]. Experimental studies showed that through the application of a stimulating effect on cells and signal ways, there is an influence on bone healing [16–19]. A few clinical studies have shown the benefit of LIPUS [15, 20, 21]. Other studies, however, have shown contrary results [22–24]. It is still unclear, whether LIPUS has a clinically relevant use in treatment of nonunions. In addition, there are no concrete conclusions concerning which patients would benefit from such a therapy. The goal of this study is to evaluate the results of LIPUS in our patient collective and in doing contribute to answering the following questions:

1. Which patients were treated with LIPUS?
2. How successful was the treatment?
3. What co-factors influenced the results?
4. Can therapy recommendations be determined?

## Methods

### Setting

The given study was conducted as a prospective, singe institution, observational cohort study. Between October 2010 and October 2013, 73 chronic nonunion patients who received treatment with EXOGEN^®^ >90 days after their last surgery, at a level 1 Trauma Centre, were included. We chose an interval of >90 days without intervention to minimize a possible influence of the surgery. In 13 patients, the desired treatment duration could not be achieved because they were treated with surgery before the end of therapy. Further exclusion criteria were possible pregnancy and age less than 18 years. One patient received LIPUS on two different long bones. Altogether 61 nonunions in 60 patient data sets have been evaluated. An experienced orthopaedic surgeon and a rehabilitation physician gave the indication for treatment with LIPUS according to EXOGEN^®^ guidelines: The fracture had to have sufficient clinical stability and there must not have been any signs of current infection. Patients received follow up regularly in our ambulant facility and were examined radiologically and clinically after 6 and 12 weeks, and after 4, 5, 6, and 12 months or completion of bone consolidation. The goal of treatment was to initiate healing of nonunions. Another experienced orthopaedic team finally examined the patients after 1 year and the analysis of the radiological examinations was completed.

### LIPUS therapy

All LIPUS patients in our study were treated with the same device (EXOGEN^®^ by Bioventus^®^, formerly Smith and Nephew^®^). Before the first treatment, the correct position of the device was controlled radiologically. After receiving the standard directions from a representative of Bioventus^®^, patients used the device for 20 min daily. In order to ensure compliance, the EXOGEN^®^ device tracked the daily length of treatment. The frequency was 1.5 MHz (±5 %), power was 30 mW/cm^2^ (±30 %), pulse duration was 200 µs (±10 %), and signal repetition was 1 kHz (±10 %).

### Evaluation of therapy

**Fig. 1:**
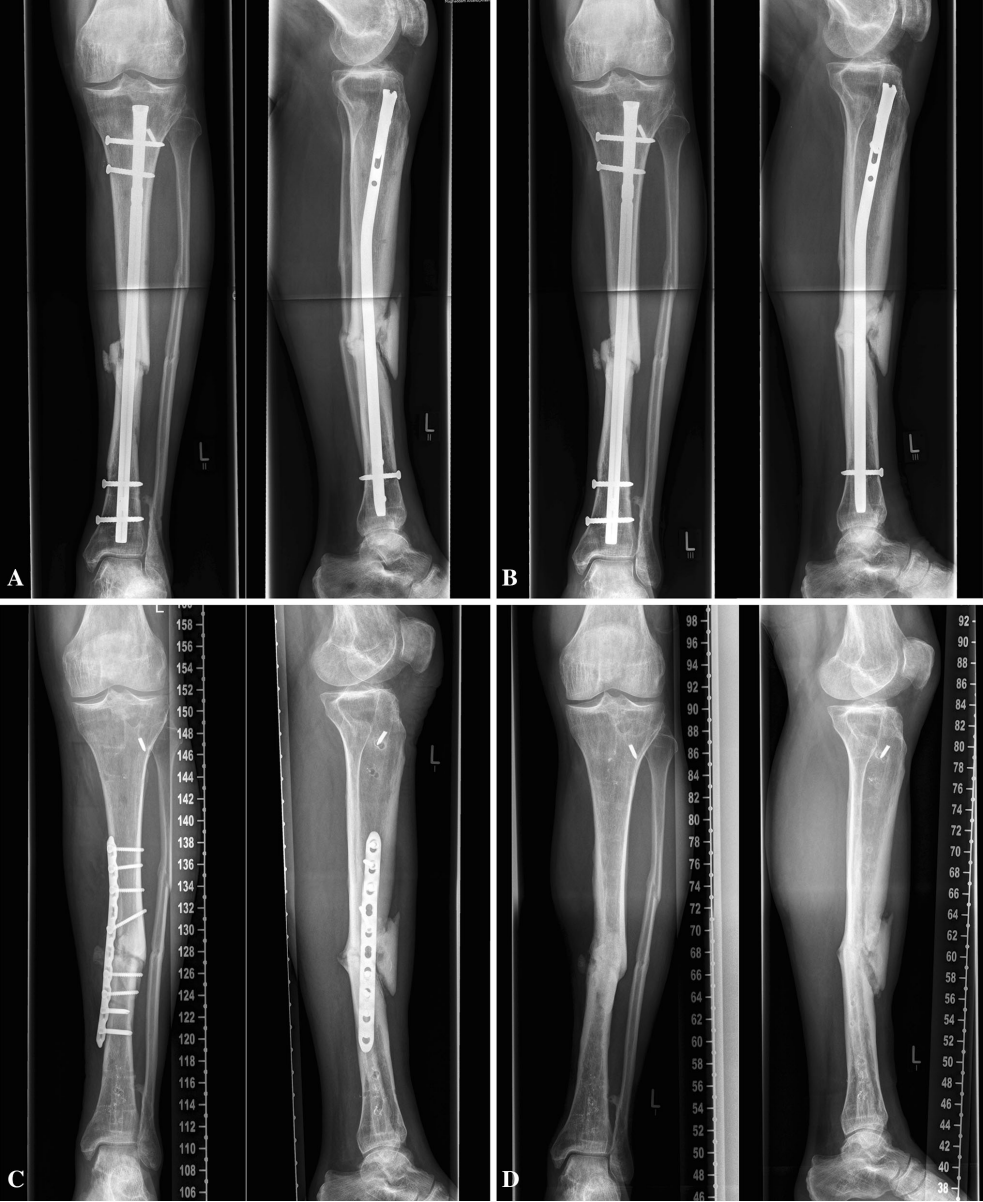
Unsuccessful LIPUS treatment. A 52-year-old male patient with a tibial nonunion, **a** start of LIPUS 1 year after trauma, **b** persisting nonunion after unsuccessful LIPUS treatment, **c** revision surgery with plate osteosynthesis including decortication, autologous spongiosa graft and BMP7 application 8 months after LIPUS, **d** healed nonunion, implant removal 8 months after revision surgery

Successful therapy was defined according to the following criteria: radiologically observed consolidation and no further surgical revision for the next year. On the other hand, therapy was unsuccessful if the nonunion did not heal and a new surgical intervention was necessary (Fig. 1).

All X-ray and CT exams performed during the study were evaluated by two orthopaedists. To assess the consolidation following criteria were used:

- Ossification in at least 3 of 4 levels.
- Secondary loosening of the implant.
- Possible secondary change in axis alignment.

The individual risk for developing a nonunion was assessed according to the presurgical nonunion score (PSN Score) (Table 1) [1]. Nonunions were classified according to non-union scoring system (NUSS) [25] (Table 2). Bone quality was evaluated according to the classification from Weber and Čech. The gap size, the Paley classification [26], the bone quality, and the bone position were evaluated.

**Table 1:** Presurgical nonunion score (PSN Score) to estimate the individual risk of patients for delayed union of long bone fractures

Localisation			
Humerus	Prox. 4 points	Diaph. 6 points	Distal 2 points
Forearm	Prox. 4 points	Diaph. 6 points	Distal 2 points
Femur	Prox. 4 points	Diaph. 6 points	Distal 8 points
Tibia	Prox. 6 points	Diaph. 8 points	Distal 4 points
Soft tissue	1° open 4 points	2° open 6 points	3° open 10 points
	Fasciotomy 4 points^a^	Previous fracture 8 points^a^	Neurological disorder 6 points^b^
Smoking	Smoker 15 points	Previous smoker 5 points	Non-smoker 0 points
Comorbidity/medication	NSAID 4 points	Bisphosphonate 6 points	Diabetes 4 points
Type 1	<10 points	Low risk	
Type 2	10–20 points	Middle risk	
Type 3	>20 points	High risk	

See Ref. [1]

^a^Affected bone

^b^Affected limb; Prox, proximal; Diaph, diaphysal

**Table 2:** Non-union scoring system

		Score^a^	Max. score
The bone			
Quality of the bone	Good	0	
	Moderate (e.g., mildly osteoporotic)	1	
	Poor (e.g., severe porosis or bone loss)	2	
	Very poor (Necrotic, appears avascular or septic)	3	3
Primary injury—open or closed fracture	Closed	0	
	Open 1° grade	1	
	Open 2°–3° A grade	3	
	Open 3° B–C grade	5	5
Number of previous interventions on this bone to procure healing	None	1	
	<2	2	
	<4	3	
	>4	4	4
Invasiveness of previous interventions	Minimally-invasive: Closed surgery (screws, k wires,…)	0	
	Internal intra-medullary (nailing)	1	
	Internal extra-medullary	2	
	Any osteosynthesis which includes bone grafting	3	3
Adequacy of primary surgery	Adequate stability	0	
	Inadequate stability	1	1
Weber and Cech group	Hypertrophic	1	
	Oligotrophic	3	
	Atrophic	5	5
Bone alignment	Anatomic alignment	0	
	Non-anatomic alignment	1	1
Bone defect—Gap	0.5–1 cm	2	
	1–3 cm	3	
	>3 cm	5	5
Soft tissues			
Status	Intact	0	
	Previous uneventful surgery, minor scarring	2	
	Previous treatment of soft tissue defect (e.g., skin loss, local flap cover, multiple incisions, compartment syndrome, old sinuses)	3	
	Previous complex treatment of soft tissue defect (e.g., free flap)	4	
	Poor vascularity: absence of distal pulses, poor capillary refill, venous insufficiency	5	
	Presence of actual skin lesion/defect (e.g., ulcer, sinus, exposed bone or plate)	6	6
The patient			
ASA Grade	1 or 2	0	
	3 or 4	1	1
Diabetes	No	0	
	Yes—well controlled (HbA1c < 10)	1	
	Yes—poorly controlled (HbA1c > 10)	2	2
Blood tests: FBC, ESR, CRP	FBC: WCC > 12	1	
	ESR > 20	1	
	CRP > 20	1	3
Clinical infection status	Clean	0	
	Previously infected or suspicion of infection	1	
	Septic	4	4
Drugs	Steroids	1	
	NSAIDs	1	2
Smoking status	No	0	
	Yes	5	5

See Ref. [25]

^a^Higher score implies more difficult to procure union

**Fig. 2:**
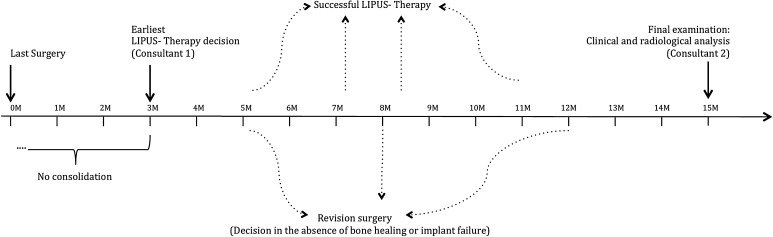
Timeline of the study protocol. Patients were treated at the earliest 3 months after surgery with LIPUS. Over the course of therapy, patients healed or needed revision surgery. Final examinations occurred 1 year after LIPUS. Final examinations followed 1 year after LIPUS. In any case, the decision for LIPUS was made by a different physician (consultant 1) than the data analysis (consultant 2)

Based on clinical and radiological evaluation as described above, patients were retrospectively divided into two groups: G1 consisted of patients with successful treatment, and G2 unsuccessful treatment (Fig. 2).

### Statistical analysis

Statistical analysis was done under the guidance of an Institute for Medical Biometrics and Informatics with SPSS 21.0 (IBM^®^ Germany GmbH) and Excel^®^ 2011 (Microsoft^®^). Depending on the scale of graphs, data were portrayed in terms of mean ± standard deviation (minimum–maximum), percentage frequency, or median with first and third quartiles. Categorical data were evaluated with the *χ*^2^ test, continuous variables with the student’s *t* test. We conducted binary logistic regression analyses. The level of significance *α* was set to 5 %.

### Ethics and source of funding

Written informed consent for participation in the study was obtained from all participants. None of the participants was underage. The study was conducted in accordance with the World Medical Association Declaration of Helsinki. The study was approved by the ethics committee of the Chamber of Medicine in Rheinland-Pfalz (837.141.08-2008 and 837.422.12-2012). There was no external funding source for this investigation.

## Results

Sixty-one nonunions in sixty patients were included in this study. All patients were available for all follow-up dates.

### Patient collective

#### Tables 3, 4, 5

The collective was mostly comprised of men (91.8 %). Patients were on average 45 ± 9.81 (18–63) years of age and had a BMI of 28.9 ± 5.4 (21.1–44.2). Twenty-six patients (42.6 %) were smokers (Table 3).

**Table 3:** Patient characteristics

Characteristic	Total		Successful treatment (G1)		Unsuccessful treatment (G2)		*p* value
	*N* = 61		*N* = 20	(32.8 %)	*N* = 41	(67.2 %)	
Gender							0.525
Male	56	(91.8 %)	19	(33.9 %)	37	(66.1 %)	
Female	5	(8.2 %)	1	(20.0 %)	4	(80.0 %)	
Age (years)	45.4	±9.81 (18–63)^a^	44.6	±11.1 (18–63)^a^	45.9	±9.1 (18–60)^a^	0.633
BMI	28.9	±5.44 (21.1–40.5)^a^	29.1	±5.5 (21.6–40.5)^a^	28.8	±5.4 (21.1–44.2)^a^	0.867
BMI > 40	5	(8.2 %)	1	(5.0 %)	4	(6.6 %)	0.714
Smoking							0.485
Smoker	26	(42.6 %)	10	(50.0 %)	16	(39.0 %)	
Previous smoker	11	(18.0 %)	2	(10.0 %)	9	(22.0 %)	
Non-smoker	24	(39.3 %)	8	(40.0 %)	16	(39.0 %)	
Diabetes Mellitus							0.115
Yes	9	(14.8 %)	5	(25.0 %)	4	(9.8 %)	
No	52	(85.2 %)	15	(75.0 %)	37	(90.2 %)	
Combined vascular risk*	3	(4.9 %)	3	(15.0 %)	0	(0.0 %)	0.011*
Smoking							0.555
Diabetes Mellitus							0.108
Arterial hypertension	12	(19.7 %)	4	(20.0 %)	8	(19.5 %)	
Hypercholesterolemia	3	(4.9 %)	1	(5.0 %)	2	(4.9 %)	
Hypothyroidism	4	(6.6 %)	1	(5.0 %)	3	(7.3 %)	
Asthma	2	(3.3 %)	1	(5.0 %)	1	(2.4 %)	
Degree of physical activity during work							0.423
Low	14	(23.0 %)	4	(20.0 %)	10	(24.4 %)	
Medium	17	(27.9 %)	4	(20.0 %)	13	(31.7 %)	
High	29	(47.5 %)	12	(60.0 %)	17	(41.5 %)	
PSN Score [1]	18.6	±9 (4–43)^a^	17.3	±8.3 (6–31)^a^	19.2	±9.2 (6–43)^a^	0.455

* Significant differences between the groups; level of significance* α *was set to 5 %

^a^Average ± standard deviation (minimum–maximum)

Overall, the patient collective had middle to high-risk profile. The average risk score according to the NUSS was 38.9 ± 10.8 (20–66) (Table 5).

Most patients received treatment of the lower extremity (Table 4). The tibia was affected in 35 patients (57.4 %), and femur in 11 (18.0 %) cases. There were 14 patients (23.0 %) with an osteitis in patients’ history before therapy. The majority of patients acquired the fracture from a fall from great heights (32.8 %), or an injury with a motorized two-wheeled vehicle (26.2 %) (Table 4). The age of the fracture from the beginning of therapy was on average 10.4 ± 8.9 (3–58) months. Patients had received an average of 3.02 ± 2.26 (1–13) operations. At the time of therapy, 37 patients (60.7 %) had osteosynthesis with a plate, 13 (21.3 %) with intramedullary nailing, and 11 (18.0 %) with an external fixateur (Table 4).

**Table 4:** Nonunion characteristics before LIPUS

Characteristic	Total		Successful treatment (G1)		Unsuccessful treatment (G2)		*p* value
	*N* = 61		*N* = 20	(32.8 %)	*N* = 41	(67.2 %)	
Treated bone							0.656
Humerus	7	(11.5 %)	3	(15.0 %)	4	(9.8 %)	
Radius	3	(4.9 %)	2	(10.0 %)	1	(2.4 %)	
Femur	11	(18.0 %)	3	(15.0 %)	8	(19.5 %)	
Tibia	35	(57.4 %)	11	(55.0 %)	24	(58.5 %)	
Others	5	(8.2 %)	1	(5.0 %)	4	(9.8 %)	
Type of fracture							0.245
Closed	44	(72.1 %)	17	(85.0 %)	27	(65.9 %)	
Open 1°	3	(4.9 %)	1	(5.0 %)	2	(4.9 %)	
Open 2°	7	(11.5 %)	2	(10.0 %)	5	(12.2 %)	
Open 3°	7	(11.5 %)	0	(0.0 %)	7	(17.1 %)	
Osteitis in patients history*	14	(23.0 %)	2	(10.0 %)	12	(29.3 %)	0.012*
Mechanism of injury							0.810
Fall from >1 m	20	(32.8 %)	8	(40.0 %)	12	(29.3 %)	
Motorcycle accident	16	(26.2 %)	5	(25.0 %)	11	(26.8 %)	
Car accident	9	(14.8 %)	2	(10.0 %)	7	(17.1 %)	
Crushing trauma	16	(26.2 %)	5	(25.0 %)	11	(26.8 %)	
Circumstances of injury							0.615
Work accident	53	(86.9 %)	18	(90.0 %)	35	(85.4 %)	
Leisure related	8	(13.1 %)	2	(10.0 %)	6	(14.6 %)	
Fracture age (months)*	10.36	±8.89 (3–58)^a^	7.2	±3.8 (3–17)^a^	11.9	±10.3 (3–58)^a^	0.011*
Number of previous surgeries	3.02	±2.26 (1–13)^a^	2.7	±2.2 (1–9)^a^	3.2	±2.3 (1–13)^a^	0.349
≥4 Surgeries	18	(29.5 %)	4	(20.0 %)	14	(34.1 %)	
Surgical treatment*							0.012*
Osteosynthesis plate*	37	(60.7 %)	17	(85.0 %)	20	(48.8 %)	0.007*
Intramedullary nailing	13	(21.3 %)	3	(15.0 %)	10	(24.4 %)	
External fixation*	11	(18.0 %)	0	(0.0 %)	11	(26.8 %)	0.011*

* Significant differences between the groups; level of significance* α *was set to 5 %

^a^Average ± standard deviation (minimum–maximum)

### Radiological characteristics

The average gap size was 0.67 ± 0.55 (0–3) cm. The bone quality was mostly moderate (34.4 %) to bad (31.1 %). Patients had hyper (39.3 %) und normo-trophic (49.2 %) nonunions. The average NUSS score for the entire collective was 38.9 ± 10.8 (20–66) (Table 5).

**Table 5:** Blinded evaluation of pre-therapeutic images

Characteristic	Total		Successful treatment (G1)		Unsuccessful treatment (G2)		*p* value
	*N* = 61		*N* = 20	(32.8 %)	*N* = 41	(67.2 %)	
Defect gap (cm)*	0.67	±0.55 (0–3)^a^	0.46	±0.29 (0–1)^a^	0.77	±0.62 (0.2–3)^a^	0.01*
Paley Classification*							0.034*
Type A	53	(86.9 %)	20	(100.0 %)	33	(80.5 %)	
Type B	8	(13.1 %)	0	(0.0 %)	8	(19.5 %)	
Quality of the bone							0.639
Good	15	(24.6 %)	6	(30.0 %)	9	(22.0 %)	
Moderate	21	(34.4 %)	6	(30.0 %)	15	(36.6 %)	
Poor	19	(31.1 %)	5	(25.0 %)	14	(34.1 %)	
Very poor	6	(9.8 %)	3	(15.0 %)	3	(7.3 %)	
Weber and Cech group							0.167
Hypterophic	24	(39.3 %)	5	(25.0 %)	19	(46.3 %)	
Normotrophic	30	(49.2 %)	13	(65.0 %)	17	(41.5 %)	
Oligotrophic	4	(6.6 %)	2	(10.0 %)	2	(4.9 %)	
Atrophic	3	(4.9 %)	0	(0.0 %)	3	(7.3 %)	
Bone alignment							0.366
Bone alignment	41	(67.2 %)	15	(75.0 %)	26	(63.4 %)	
Non-anatomic alignment	20	(32.8 %)	5	(25.0 %)	15	(36.6 %)	
NUSS*	38.9	±10.8 (20–66)^a^	34.7	±8.9 (24–54)^a^	41.0	±11.1 (24–66)^a^	0.034*

* Significant differences between the groups; level of significance* α *was set to 5 %

^a^Average ± standard deviation (minimum–maximum)

### Results of LIPUS

Twenty cases (32.8 %) had a successful LIPUS therapy (G1), and 41 cases (67.2 %) had to receive further surgery (G2). In patients with healed nonunions there was an average time of consolidation of 5.3 ± 1.9 months. The average duration of therapy was 5.5 ± 1.8 (2–9) months.

The time from beginning LIPUS therapy to achievement of full weight bearing was on average 7.5 ± 5.6 (1–23) months. In 15 cases (24.6 %), full weight bearing was not reached at the end of the study (Table 6).

**Table 6:** Outcome of LIPUS-therapy

Characteristic	Total		Successful treatment (G1)		Unsuccessful treatment (G2)		*p* value
	*N* = 61		*N* = 20	(32.8 %)	*N* = 41	(67.2 %)	
Full weight-bearing (months)^b,^*	7.5	±5.6 (1–23)^a^	5.4	±3.9 (1–14)^a^	9.1	±6.1 (1–23)^a^	0.037*
Full weight-bearing not achieved	15	(24.6 %)	2	(10.0 %)	13	(31.7 %)	0.065
Full weight-bearing possible before LIPUS- therapy	7	(11.5 %)	1	(5.0 %)	6	(15.0 %)	0.144
Time out of work (months)*	16.2	±8.3 (1.3–36)^a^	12.2	±8.1 (1.3–36)^a^	19.0	±7.3 (5–36)^a^	0.017*
Disability after therapy	24	(39.3 %)	5	(25.0 %)	19	(46.3 %)	0.122
Subjective evaluation of pain*							0.004*
Improvement	5	(8.2 %)	5	(25.0 %)	0	(0.0 %)	
Minimal improvement	13	(21.3 %)	5	(25.0 %)	8	(19.5 %)	
No change	37	(60.7 %)	8	(40.0 %)	29	(70.7 %)	
Worsening	1	(1.6 %)	0	(0.0 %)	1	(2.4 %)	
No pain	5	(8.2 %)	2	(10.0 %)	3	(7.3 %)	

* Significant differences between the groups; level of significance* α *was set to 5 %

^a^Average ± standard deviation (minimum–maximum)

^b^Surgical intervention in unsuccessful LIPUS-therapy

All patients were disabled after accident. On average, the duration of disability was 16.2 ± 8.3 (1.3–36) months. Twenty-four cases (39.3 %) were disabled at the end of the study.

Upon completion of LIPUS, five patients (8.2 %) reported an improvement in pain, 13 (21.3 %) minimal improvement, 37 (60.7 %) no improvement, and one patient (1.6 %) said that pain had become more severe (Table 6).

### Complication

One patient with a history of osteitis developed an abscess in the fourth week of LIPUS therapy that needed to be treated surgically.

### Comparison of groups

#### Tables 3, 4, 5, 6

**Fig. 3:**
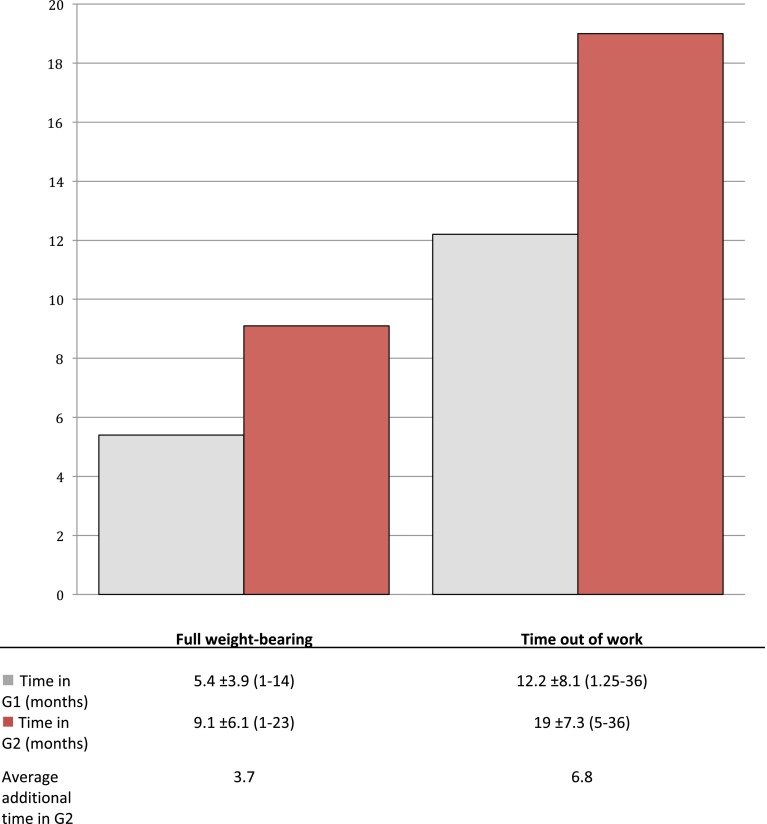
Delay in time of treatment in G2. Patients from the unsuccessful group had an average additional time to full weight-bearing of 3.7 months and an average additional time out of work of 6.8 months; mean ± standard deviation (minimum–maximum)

The duration until full weight bearing was achieved was significantly different between the groups. It was 5.4 ± 3.9 (1–14) months in the successful group and 9.1 ± 6.1 (1–23) months in the unsuccessful group (*p* = 0.037). In G1, two patients (10 %) did not reach full weight bearing; in G2 it was 13 (31.7 %) (*p* = 0.065) (Table 6). On average, patients in group 2 reached full weight bearing 3.7 months later than group 1 (Fig. 3).

Also the length of disability was significantly different between groups. In the consolidated group it was 12.2 ± 8.1 (1.3–36) months, in the non-consolidated group 19 ± 7.3 (5–36) (*p* = 0.017).

In G1, 5 patients (25 %) stayed disabled after therapy, in G2 19 (46.3 %) (*p* = 0.122) (Table 6). Patients in G2 returned to work 6.8 months later than G1 (Fig. 3).

**Fig. 4:**
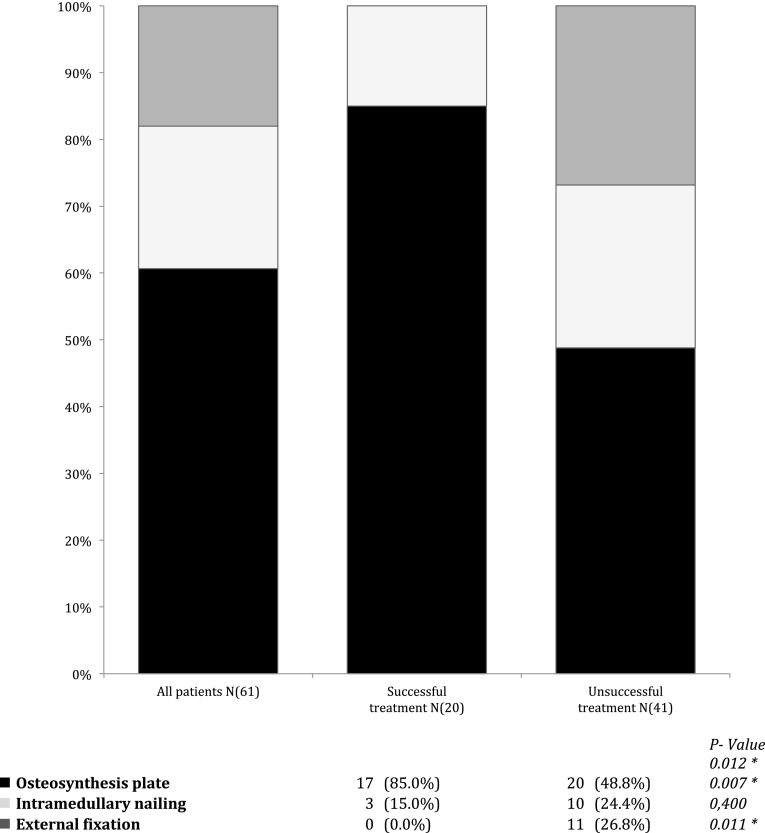
Surgical treatment before LIPUS Therapy. *Asterisk* indicates a significant difference in operative treatment between the successful and unsuccessful group

There was a difference in the standardized subjective evaluation of therapy between G1 and G2. Patients in G1 reported significantly more lessening of pain (*p* = 0.004). The length of treatment did not significantly differ between groups (G1: 5.3 ± 1.9 months; G2: 5.6 ± 1.8 months). There were no significant differences amongst groups regarding patient characteristics (Table 3). In G1, there were only 2 cases of previous infection; G2 had 12 (*p* = 0.012) (Table 4). The binary logistic regression model had the following results: Patients with past osteitis had a 4.6- (95 % CI 1.5–14.7) fold higher relative risk of treatment failure compared to patients without infection in prehistory. The age of the fracture at the beginning of treatment differed between G1 and G2 significantly [G1: 7.2 ± 3.8 (3–17) months; G2: 11.9 ± 10.2 (3–58) months] (*p* = 0.011). Furthermore we detected a significant difference between G1 and G2 in the type of osteosynthesis used at the time of the therapy (*p* = 0.012) (Fig. 4). Consolidated patients were more frequently treated with plate osteosynthesis (*p* = 0.007), non-consolidated with external fixation (*p* = 0.011). The binary logistic regression model had the following results: Nonunions treated with plate osteosynthesis had a 6.0- (95 % CI 1.5–23.5) fold higher relative chance of success of LIPUS therapy compared to treatment with other surgical procedures.

**Fig. 5:**
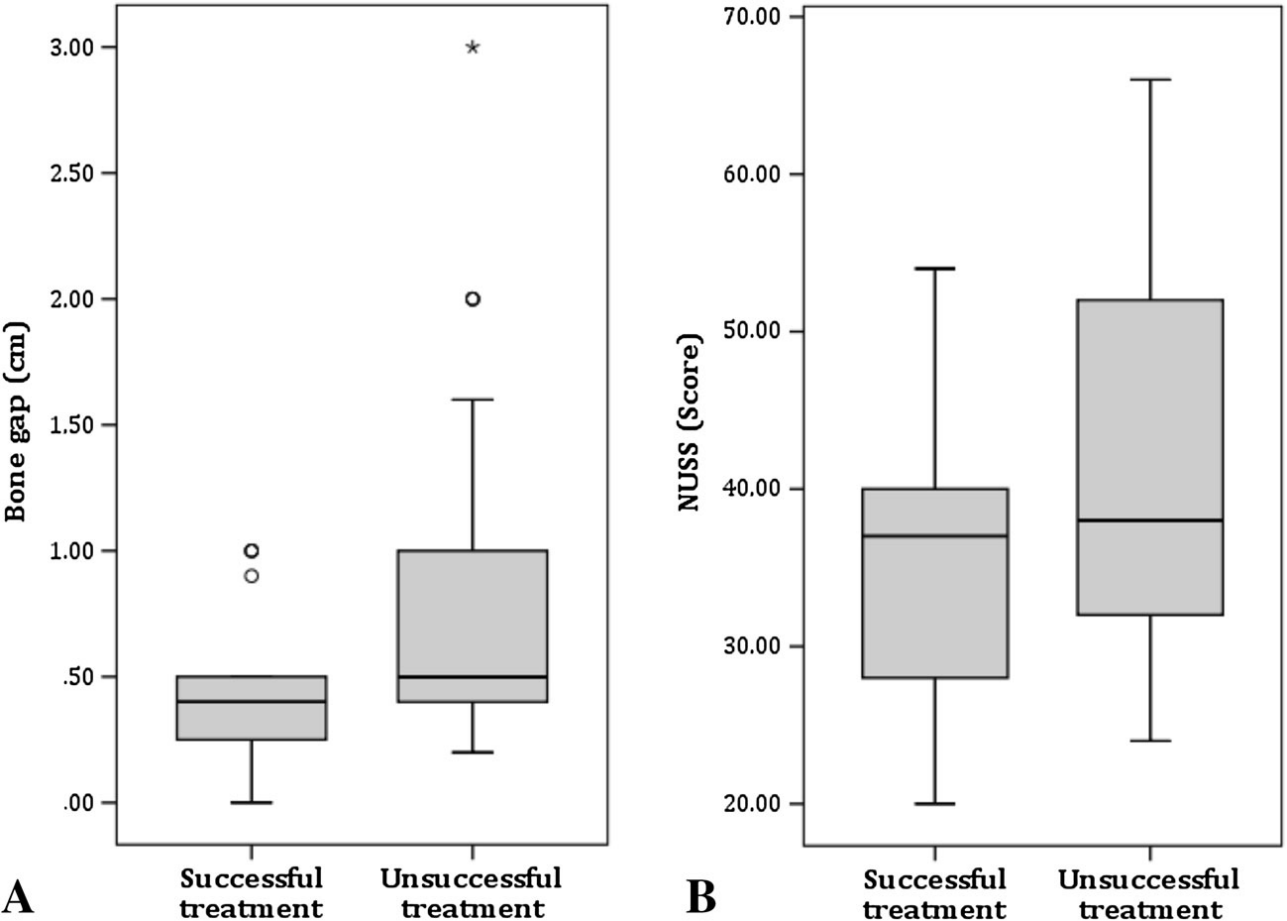
Distribution of gap size and NUSS Score. **a** Distribution of gap size in cm: Patients with unsuccessful treatment showed a significantly larger bone gap (*p* = 0.01). **b** Distribution of NUSS Scores: Patients with unsuccessful treatment showed a significantly higher NUSS Score (*p* = 0.034)

The average gap size before therapy in the successful group was 0.46 ± 0.29 (0–1) cm, in the unsuccessful group, 0.77 ± 0.62 (0.2–3) cm (*p* = 0.01) (Table 5; Fig. 5a).

Overall, G1 and G2 differed in the NUSS Score of nonunion (*p* = 0.034). The NUSS Score in the successful group was 34.7 ± 8.9 (24–54) points; in the unsuccessful group it was 41 ± 11 (24–66) (Table 5; Fig. 5b).

## Discussion

In this observational study, 61 nonunions were treated with LIPUS. All patients took part in follow-up. The LIPUS treatment was only successful in 20 nonunions (32.8 %) of the observed patients. The strength of this study is the low dropout rate and the regular follow-up scheme. This allowed one to observe the course of the healing process in all patients over an entire year. Two Different physician teams did the indication and analysis of LIPUS to avoid observer bias. No author had any potential financial or personal competing interests that might have influenced the results. The weakness of the study was the lack of a prospective comparison group.

### Success rate of LIPUS

The primary therapy goal was to heal nonunions through LIPUS therapy. This was observed in only 32.8 % of the cases. The average time to healing was 5.3 ± 1.9 (2–7) months, which is comparable to results from former studies [27].

In the literature, various rates of healing for LIPUS that range between 55 and 88 % have been reported [14, 15, 20, 21, 27–29]. Because of the high healing rates some authors see LIPUS as an effective alternative to surgery [27, 29]. The most recent study with a total number of 767 analysed chronic nonunions from Zura et al. showed a healing rate of 86.2 %. The authors concluded that LIPUS could be an effective alternative to surgical revision in the treatment of nonunions [27]. However, this conclusion shows some weaknesses that should be taken into consideration.

The indication criteria for surgical treatment of nonunions differ greatly from those for LIPUS. For example, LIPUS treatment needs compulsively mechanical stability of the nonunion site [15]. In addition, a small gap size is important for LIPUS treatment success [15]. In the presented study we found different other factors, which may affect the outcome, like a previous infection of the bone or an overall challenging nonunion (high NUSS Score). Because nonunions that did not meet the LIPUS treatment criteria have been treated surgically, a positive selection of the patient collective, which might have biased the results, can be assumed. To make a better statement on a possible alternative to surgery, studies should classify nonunions more differentiated and stratify the risk by supplying more detailed information on the nonunions characteristics or using scoring systems, like the NUSS. Furthermore, in this study only 91 cases have been chronic nonunion patients who received LIPUS >90 days after their last surgery [27]. Therefore, a possible influence of the last surgery cannot be excluded. In the presented study we only included patients who did not receive any intervention for 90 days to minimize a possible bias. Another problem might arise from the fact that there has not been a standardized follow-up scheme in this study. This makes it difficult to assess the healing process in a standardized way. All together the recent study from Zura et al. presents a highly selected patient collective and might result in misleading conclusions [27].

In the only randomized controlled study on LIPUS treatment including 101 chronic nonunion patients, 51 patients were treated with an EXOGEN^®^ device, and 50 patients with a sham device. There was no significant difference in the rate of healing between intervention and placebo groups: the rate of healing for the EXOGEN^®^ group was 65 % (33/51), and placebo 46 % (23/50) [20].

The rate of healing in our collective was 32.8 %, below that of 55–88 % given in the literature [14, 15, 20, 21, 27, 28]. One possible reason for the low healing rate is the high number of difficult nonunions in our patient collective. The patients had an elevated average NUSS Score of 38.9 ± 10.8 (20–66). Another possible explanation for the poor results in our collective is that LIPUS was presented as an alternative for many patients after a string of unsuccessful operative interventions. On average, our collective had had three surgeries, and almost one-third of the collective (29.5 %) over four times. In a study from 2001 with a rate of healing of 86 % patients had only received an average of 1.4 previous operations [29]. Through the high number of failed previous operations in our collective, it can be assumed that a negative selection took place.

### Treatment Indication

In our study, patients with small gap sizes and a low NUSS score benefited most from LIPUS treatment (Table 5). Patients with general risk factors such as advanced age or high BMI, as well as diabetes mellitus have shown no significant difference in treatment outcome (Table 3). Patients had a high risk of developing nonunions apparent by looking at the PSN Score showing that 75.4 % had a middle or high risk of developing a nonunion. Therefor we assume that LIPUS treatment should only be considered as a treatment option for a highly selected patient collective with low risk factors.

### Complications

In the period of therapy, we documented the formation of an abscess in one patient. One explanation is that it might have been coincidence. But former studies have shown a connection between atrophic nonunions and bone infection [30]. Therefor another possible interpretation might be that the biofilm of the implant has been mobilized by the ultrasound. This leads to the assumption that the use of LIPUS might be seen critically in patients after bone infection. A study in the US involving 55000 EXOGEN^®^ systems spanning 1 year of therapy showed that there was only skin irritation in three cases and chest pain in one patient, which may have been due to an interaction with a pacemaker [31]. Nevertheless, we believe that studies on LIPUS should not neglect possible complications due to the danger of the resurgence of a past infection.

### Comparison of groups

Our study took the time to full weight bearing as well as the length of disability as functional outcome parameters. In unsuccessfully treated patients, full weight bearing took on average 3.7 months longer, the time to returning to work 6.8 months (Fig. 3).

The standardized subjective questionnaire showed that most patients were unsatisfied with the LIPUS therapy. More than half of patients (70.5 %) did not find LIPUS helpful in the standardized questionnaire (Table 6). Assumingly, this is due to the poor treatment result.

There was a significant difference in the age of the fracture in G1 and G2. Jingushi et al. found in their analysis of 72 patients treated with LIPUS that delayed fracture healing and nonunions should be treated within the first 6 months after the last operation [21]. Authors stated that 90 % of fractures healed in patients in the first 6 months. If, however, LIPUS took place after the 6th month post-op, the success rate sank to 65 %. Nonunions heal in the first 6 months after surgical treatment anyway without further intervention in most cases, as shown in a study from 2009 with postoperative rates of healing between 80 and 100 % [32]. For this reason, it does not seem meaningful to treat all patients with LIPUS.

We were able to show that plate osteosynthesis showed the best results, external fixation the worst (Fig. 4). This suggests that LIPUS is more appropriate for certain types of osteosynthesis. In this study we found that patients treated with plate osteosynthesis had a 6.0- (95 % CI 1.5–23.5) fold higher relative chance of success than patients treated with other surgeries. A Japanese research group found that patients treated with intramedullary nailing had a worse outcome with a relative risk of 4.5 (95 % CI 1.69–12.00) [14].

Watanabe et al. postulated that there are three key radiological factors that can influence the results of LIPUS: the gap size, the stability of the osteosynthesis, and the fracture classification [14]. In this prospective cohort study, 101 delayed fractures and 50 nonunions have been examined. The rate of consolidation was 68.0 % (34/50) for patients with nonunion and 74.3 % (75/101) for patients with delayed fractures healing.

Authors saw the gap size as a limiting factor for therapy. The results of our analysis are consistent with the literature, as we also found that the gap size influenced therapy results. A gap size of over 1 cm seems to be a contraindication for LIPUS [14, 15] (Table 5; Fig. 5a).

In our study, we found signs that previous infection influences LIPUS negatively (Table 4). Also the documented abscess may have been due to the influence of LIPUS. We found that patients with past osteitis had a 4.6- (95 % CI 1.5–14.7) fold higher relative risk of treatment failure compared to patients without infection in prehistory. An association between LIPUS and previous infection of the bone has not yet been examined in previous studies. This aspect should be looked at in future studies. A summary of the discussed therapy related cofactors is listed in Table 7.

**Table 7:** Therapy related cofactors

Positive factors	Negative factors	*p* value
Plate osteosynthesis	External fixation	0.012*
Small defect	Large defect (>1 cm)	0.034**
No infection in patients history	Osteitis in patients history	0.012*
Fracture age <6 months	Fracture age >6 months	0.011**
Low NUSS Score	High NUSS Score	0.034**

Listed cofactors show significant differences between the groups and should be considered in decision-making

* *χ* ^2^ test

** Student’s* t* test

In the literature it is has been suggested that LIPUS has a biological effect [16, 17]. It remains unclear whether this effect is clinically relevant or not [33]. However, it is uncertain if spontaneous healing is responsible for the documented success. A possible use of LIPUS is supportive treatment of nonunions in risk patients, but clinically and radiologically it is difficult to evaluate the use of LIPUS. An objective method for testing its effect could be the measurement and analysis of the serum cytokine profile that is involved in bone metabolism, e.g., TGFß-1, VEGF, PDGF, or bFGF [34], which can be evaluated objectively and have a small tendency towards bias [34].

## Conclusion

In our patient collective, 32.8 % of patients treated with LIPUS showed healing, despite strictly following indication criteria for EXOGEN^®^. Following criteria should be considered prior to LIPUS therapy: mechanical stability, small gap sizes (<1 cm), short interval to treatment after injury, and the absence of acute or past infection. Such a collective actually has a high tendency towards spontaneous healing. In the remainder of patients, LIPUS lead to a significant extension of therapy and disability.

Our results show that LIPUS should not be seen as a generally accepted alternative therapy in the treatment of long bone nonunions. Furthermore its indications differ considerably from surgical ones. Therefore, LIPUS should be evaluated on a case-to-case basis.
